# Applications in the search for genomic selection signatures in fish

**DOI:** 10.3389/fgene.2014.00458

**Published:** 2015-01-14

**Authors:** María E. López, Roberto Neira, José M. Yáñez

**Affiliations:** ^1^Faculty of Agricultural Sciences, University of ChileSantiago, Chile; ^2^Aquainnovo, Puerto MonttChile; ^3^Faculty of Veterinary and Animal Sciences, University of ChileSantiago, Chile

**Keywords:** fish, selection, domestication, single nucleotide polymorphisms, genome

## Abstract

Selection signatures are genomic regions harboring DNA sequences functionally involved in the genetic variation of traits subject to selection. Selection signatures have been intensively studied in recent years because of their relevance to evolutionary biology and their potential association with genes that control phenotypes of interest in wild and domestic populations. Selection signature research in fish has been confined to a smaller scale, due in part to the relatively recent domestication of fish species and limited genomic resources such as molecular markers, genetic mapping, DNA sequences, and reference genomes. However, recent genomic technology advances are paving the way for more studies that may contribute to the knowledge of genomic regions underlying phenotypes of biological and productive interest in fish.

## INTRODUCTION

Selection signatures are genomic regions that harbor DNA sequences involved in genetic variation of traits subject to natural or artificial selection (Qanbari et al., 2012). Currently, due to advances in genomic technologies and statistical methods, such signatures can be identified in the genomes of various species.

Most studies in this field of genetics are based on the concept of *hitchhiking*, which suggests that selection affects the genome at a specific region, leaving “signatures” around the selected gene(s) (Smith and Haigh, 1974). Specifically, the hitchhiking theory focuses on the spread of new variants in a population due to selection for their favorable effects (Przeworski, 2002; Kim and Nielsen, 2004). Selection involving alleles from the population’s standing genetic variation produces specific and detectable DNA sequence patterns (Hermisson and Pennings, 2005).

The search for these molecular signatures has been the subject of intense research in recent years in both domesticated and wild populations of plants and animals, as well as in humans. These studies have been motivated by two main objectives: (1) a strong interest in the evolutionary past of the species and basic molecular mechanisms governing this evolution and (2) the expectation of an association between these genomic regions and biological functions or phenotypes of interest, since these regions should have some functional or adaptive importance underlying their selection (Nielsen et al., 2007). These studies are possible due to the development of various methods aimed at detecting selection at the molecular level in population samples. Information on allelic frequencies or haplotype patterns segregated in the population can be used to identify signatures, since selection modifies the patterns of genetic variation expected under the neutral theory of molecular evolution.

Most studies in domesticated populations have focused on detecting relatively old selection signatures dating back hundreds or thousands of generations, e.g., (Flori et al., 2009), with few studies on genetic changes during early domestication stages (Trut et al., 2009).

Certain fish species provide unique models for studying the effects of selection and domestication, as their populations were domesticated recently and are available as both wild and domesticated populations simultaneously.

In this article we present different aspects involved in studying selection signatures at a genomic level in different species and discuss about the potential application of these studies in fish populations to unravel recent selection and domestication processes in these species.

## IDENTIFICATION OF LOCI ASSOCIATED WITH TRAITS OF INTEREST

The search for genes controlling phenotypic variation can be performed in two different ways. First, the “top–down” approach which begins with knowledge of the phenotype of interest and uses genetic analysis to identify genes or causal regions. These approaches include candidate gene studies, identification of Quantitative trait loci (QTLs) and association mapping. These studies have certain limitations, including the need for an *a priori* hypothesis about which genes underlie the trait of interest, information about family relationship between individuals, as well as, access to a large number of relatives with phenotypic records (Gu et al., 2009). Second, the “bottom–up” approach, in contrast, begins with genomic information and involves statistical evaluation of molecular information to identify regions subject to selection (Ross-Ibarra et al., 2007). This approach searches for patterns of linkage disequilibrium, genetic differentiation, or frequency spectrum that are inconsistent with the neutral evolution model to identify selection signatures (Qanbari et al., 2010). Recent advances in genomics provide a new paradigm for the “bottom–up” strategy concerning population genomics, a discipline that infers genetic and evolutionary parameters of a population based on datasets from the whole genome (Black et al., 2001).

In this context, population genomics relies on two basic principles or assumptions. First, neutral loci will be equally affected by demographic effects and by the evolutionary history of the population. Second, loci under selection will tend to behave distinctively, revealing atypical variation patterns (Luikart et al., 2003).

## MODELS OF SELECTION

Natural selection can be defined as the differential contribution of genetic variation to future generations (Aquadro et al., 2001) due to differential reproduction of some phenotypes/genotypes over others under prevailing environmental conditions at a given time (Futuyma, 1998). It is the driving force behind Darwinian evolution and can be subdivided into different types, depending on the evolutionary outcome (Hurst, 2009).

Directional selection tends to decrease variation *within* a population but may increase or decrease variation *among* populations. Positive selection is a type of directional selection that favors alleles that increase fitness of individuals. When directional selection eliminates unfavorable mutations, it is called purifying selection (also known as negative selection).

Diversifying (or disruptive) selection favors variety and benefits individuals with extreme phenotypes over intermediate. In this type of selection, the propagation of an allele never reaches fixation, and therefore it may occur when an allele is initially subject to positive selection, and then negative selection when the frequency becomes too high (Nielsen, 2005).

Balanced selection, which helps to maintain an equilibrium point at which both alleles remain in the population, has several forms, including frequency-dependent selection and overdominance, which occurs when the heterozygote has the higher biological fitness, and therefore variability is maintained in the population (Nielsen, 2005).

## SELECTION SIGNATURES

In the classic “hitchhiking” scenario, first described by Smith and Haigh (1974), a new allelic variant that represents a favorable adaptive substitution originates within the population as a new mutation, and its frequency increases as a result of constant selection pressure. When a favorable allele is selected, and its frequency increases to fixation in a population, genetic variation in the surrounding DNA segment is altered; that is, the increased frequency of the selected allele also produces increased frequency of closely-linked alleles (Pennings and Hermisson, 2006).

The ancestral variation, i.e., genetic variation present in a population prior to a selection process, is maintained only if recombination during this phase disrupts the association between an adjacent locus and the selected site. The resulting pattern of such a selective event is a strong reduction in genetic variation around the selected site, known as a “*hard sweep,”* which corresponds to the classic selective sweep (Pritchard et al., 2010).

There is a second scenario in which an adaptive substitution involves multiple copies of a favorable allele in the population. This may occur for two reasons. First, when an adaptation arises from genetic variation, many copies of the favorable allele may be present in the population. Fixation of this allele may involve descendants of more than one of these copies. Second, a favorable allele can be introduced in the population by recurrent mutation or migration during a selection phase, and again, several descendants of independent origin may contribute to the allelic fixation. In both cases, different alleles of loci adjacent to any such favorable copies will be retained in the population, resulting in different haplotypes (Pennings and Hermisson, 2006).

Selection signatures involving descendants of more than one copy of the selected allele and, therefore with different haplotypes at closely-linked sites, are called “soft sweeps.” This type of selection signature results in different haplotype patterns than the “*hard sweeps*” described above and it is more difficult to detect as it only produces a slight reduction in the levels of adjacent polymorphisms (Cutter and Payseur, 2013).

Furthermore, when adaptation occurs by polygenic selection, it induces an increase in the allelic frequency of several loci which have a favorable effect on a particular phenotype; however, these polygenic alleles do not necessary achieve fixation, and the resulting haplotype pattern corresponds to several partial selection signatures or multiple “partial sweeps” (Pritchard et al., 2010).

Finally, when purifying or negative selection reduces the frequency or eliminates a deleterious allele, the genetic diversity at linked loci also decreases, which is known as “*background selection*” (Charlesworth, 1993).

**FIGURE 1:**
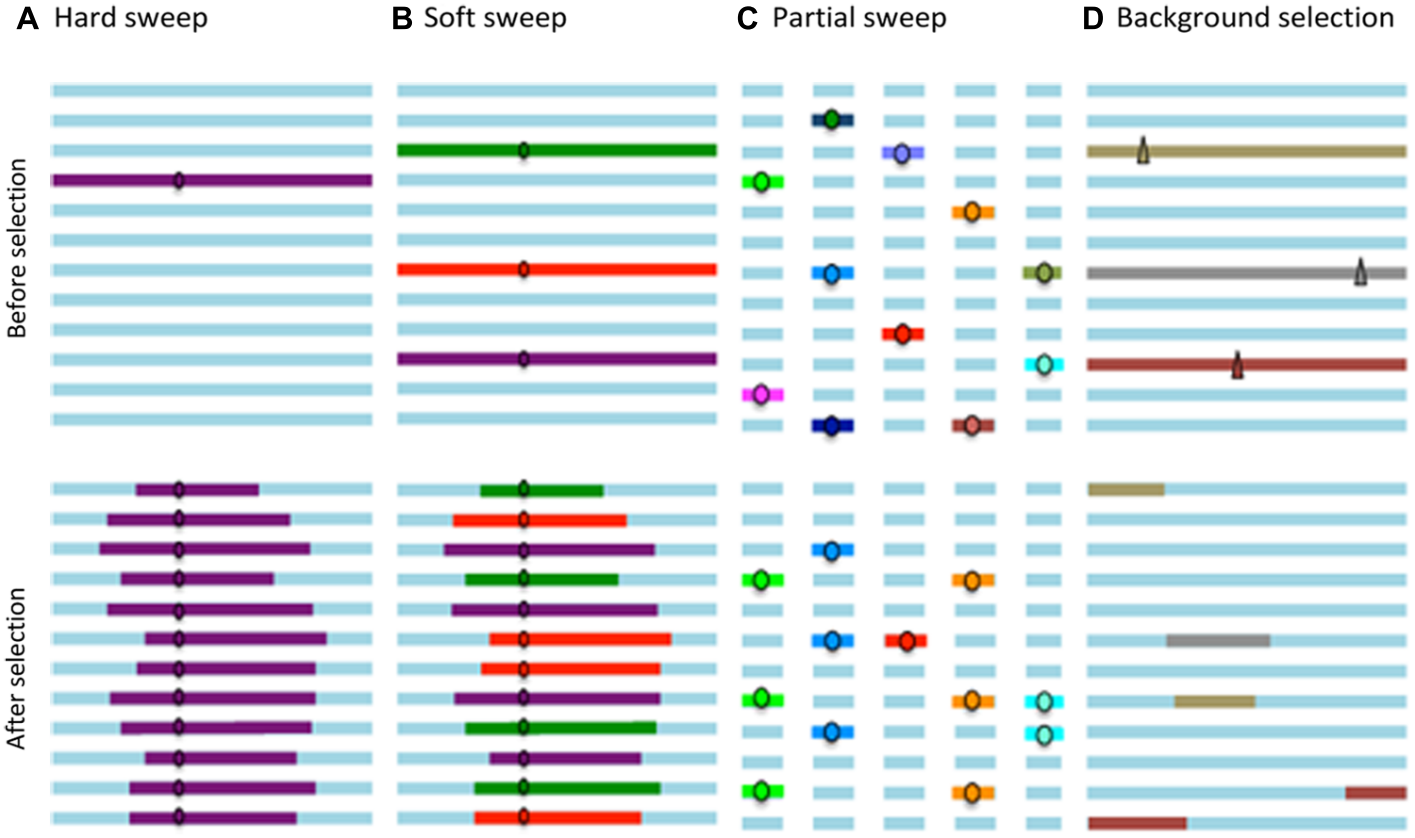
**The horizontal lines represent the haplotypes in a population; the circles favorable alleles; and the triangles deleterious alleles. (A)** Hard sweeps involve fixation of a new favorable variant. **(B)** Soft sweeps involve fixation of equivalent alleles from different genetic backgrounds and therefore result in different haplotypes. **(C)** Partial sweeps involve increased allele frequency at several loci, without reaching fixation. **(D)** Background selection eliminates deleterious variants and linked loci.

**Figure 1** schematically summarizes the patterns caused by “*hard sweeps*,” “*soft sweeps*,” and “partial sweeps” that correspond to selective events for favorable variants in a population, as well as the pattern produced by “*background selection.”*

## DOMESTICATION AND RECENT ARTIFICIAL SELECTION IN FISH

Domestication is the process by which various species have been adapted to a captive environment by humans. Such adaptation is accomplished through systematic breeding over generations and is characterized by changes in behavior, morphology, and physiology, as well as adaptive genetic changes caused by artificial and natural selection (Price, 1999).

In fish, domestication occurred very recently as compared to other land animals. One theory to explain the late domestication of aquatic species suggests that, due to the high fertility of these species, a small number of broodstock were required to obtain a sufficiently large progeny in subsequent generations. After a few generations, the inbreeding depression increases considerably; therefore, fitness and productive behavior decrease. As a result, fish farmers were forced to repeatedly take new broodstock from the wild environment, interrupting the continuity of domestication and breeding (Gjedrem et al., 2012). For this reason, aquaculture has lagged behind land animal and plant culture in the use of breeding to enhance biological production efficiency.

On the other hand, it is estimated that less than 10% of aquaculture production is based on genetically improved stocks (Gjedrem, 2012), although the annual genetic gains reported for aquaculture species are substantially higher than for land animals. For example, selection response reported for grow-related traits are even higher than 10% in fish populations, which can substantially enhance aquaculture production by selective breeding (Gjedrem et al., 2012). Recent high selective pressures in farmed fish populations may have shaped genome variation in regions harboring causative mutations of selected traits. The identification of these regions may help in the understanding of the effect of selection events and identification of genetic variants involved in phenotypic variation in fish populations.

### EXAMPLES OF DOMESTICATION AND BREEDING PROGRAMS IN AQUATIC SPECIES

The fish belonging to the family *Ciprinidae* are most likely the first fish species to be domesticated. For instance, the goldfish (*Carrassius auratus*) is an ornamental fish, believed to have been domesticated in China before the XVI century and later taken to Japan and Europe (Purdom, 1993). Another important group of ornamental fish is the koi carp, a variety derived from common carp (*Cyprinus carpio*) and mainly cultivated in Japan. The large variety of colors and forms among koi carp resulted from directed selection and crossbreeding (Gjedrem, 2005).

There is evidence in common carp showing a large response to selection for furunculosis survival rates (Schaperclaus, 1962). In 1987, Ilyassov (1987) showed results from 4 to 5 generations of selection in this species for resistance to dropsy disease, which increased survival by 30–40% as compared to unselected carps. On the other hand, selection responses reported for growth rate in rohu carp (*Labeo rohita*) have been particularly high, reaching almost a 30% per generation (Gjedrem, 2012).

Furthermore, salmonid species are the most intensively selected fish populations. In this regard, the rainbow trout (*Oncorhynchus mykiss*) has a long history of domestication and breeding in the United States, Norway, Finland, and Denmark (McAndrew and Napier, 2011). In 1932, investigators began to select individuals to improve growth rate, number of eggs, and characteristics of sexual maturity (Donaldson and Olson, 1957). Currently there are 13 breeding programs worldwide aimed at improving growth rate, age at sexual maturity, fillet quality, and disease resistance in this species (Rye et al., 2010).

In the case of Atlantic salmon (*Salmo salar*), breeding programs exist in Norway, Scotland, Ireland, Australia, and Chile (Norris et al., 1999; Metcalfe et al., 2003; Glover et al., 2009; Dominik et al., 2010; Rye et al., 2010; McAndrew and Napier, 2011). Several traits of commercial interest such as growth, sexual maturity, meat quality, and disease resistance have been incorporated into breeding objectives. Furthermore, findings from genomic technologies have been incorporated into these breeding programs, for example, the use of QTLs to assist selection for resistance against the viral disease named infectious pancreatic necrosis (Houston et al., 2008; Moen et al., 2009).

Among Pacific salmon, the chinook salmon(*Oncorhynchu stshawytscha*) originating in British Columbia (BC), Canada was one the first species of salmons to be domesticated (Kim et al., 2004). Currently, its farming is limited and there are two breeding programs in operations (Rye et al., 2010). Moreover, genetic improvement programs for coho salmon (*Oncorhynchus kisutch*) have been successful in selecting for harvest weight and early spawning, with selection responses of about 10% per generation (Neira et al., 2006).

Tilapias are the second-most important group of cultivated fish in the world. The dominant species is the Nile tilapia (*Oreochromis niloticus*); however, other species of the genus *Oreochromis* (Neira, 2010) are also cultivated. The GIFT (Genetic Improvement of Farmed Tilapias) program, begun in 1987 in the Philippines, systematically compared wild and commercial strains in various aquatic environments and established a family-based selection system to improve growth rate (Eknath et al., 1993). The program is currently managed by the World Fish Center in Malaysia and genetic gains for growth-related traits are among 10–15% (Ponzoni et al., 2011).

Breeding programs have recently been established for other important species such as, sea bass (*Dicentrarchus labrax*; Vandeputte et al., 2009), sea bream (*Sparus aurata*), turbot (*Scophthalmus maximus*), Atlantic cod (*Gadus morhua*; Glover et al., 2011), halibut (Glover et al., 2007), and tuna (Owen, 2011).

All of these domestication and artificial selection processes shape the genomes of cultured fish populations, resulting in selection signatures that could potentially be identified using molecular and statistical methods.

## APPROACHES USED FOR DETECTING SELECTION SIGNATURES

When a new allelic variant that does not affect the fitness of individuals originates in a population, it is not affected by natural selection and is said to be neutral. Statistical tests aimed at testing a neutral evolution model can be divided into three main classes: (1) tests based on polymorphisms within species; (2) tests based on the differences between species; and (3) tests that use information within and between species. A description of these three approaches is given below.

### TESTS BASED ON POLYMORPHISMS WITHIN SPECIES

#### Frequency spectrum

The frequency spectrum is defined as the allele frequency distribution of a large number of independent loci in a given sample (Nielsen, 2005; Vogl and Clemente, 2012). Deviations from expectations of the neutral model (no selection, recombination, population subdivision, or changes in the effective population size) could be indicative of selection: purifying or negative selection tends to increase the fraction of mutations segregating at low frequencies, while positive selection increases the number of alleles observed at high frequencies (Hurst, 2009).

Many tests for detecting selection signatures are based on information provided by the frequency spectrum obtained from DNA sequence data. One of the most commonly used is the Tajima’s (1989) *D* test, which compares two measures of genetic variation (θ). The first is obtained from the average of nucleotide differences between pairs of sequences, and the second is the total number of segregating sites (Nielsen, 2005). If the difference between these two measures is greater than expected under neutral evolution, this model is rejected. Other tests have incorporated phylogenetic information in order to estimate the direction of change and increase power to detect deviations from the null hypothesis of the neutral model (Perfectti et al., 2009). One such test is that of Fu and Li (1993), which also calculates a statistic based on the comparison of two genetic variation estimates, adding phylogenic information. For example, a related species may be added as an outgroup, such as the inclusion of the chimpanzee in an analysis of human genetic variation (Nielsen, 2005). Likewise, Fay and Wu (2000) developed a test based on the concept that the frequency spectrum expected under neutrality must be enriched with mutations at low frequencies, and that therefore, mutations at high frequencies are atypical.

Researchers have used this approach to detect selection signatures in several species. In humans, for example, evidence of selection has been found in genes related to the immune system and social behavior (Sabeti et al., 2002; Williamson, 2007). In other species such as chickens, it has been possible to identify genomic regions related to production-related traits such as eggshell hardness and immune system characteristics (Qanbari et al., 2012).

#### Linkage disequilibrium (LD) and haplotype structure

Linkage disequilibrium (LD) refers to the non-random association of alleles at two or more loci. That is, if two alleles at two loci segregate together in greater proportion than expected by chance, it is said that these loci are in linkage disequilibrium. This measure has been widely used to study various demographic events and evolutionary processes in plants and animals, such as breeding systems, patterns of geographic subdivision, events of natural, and artificial selection, gene conversion, mutation, and other forces that can cause changes in gene frequency (Slatkin, 2008). The LD is affected by different evolutionary factors, including recombination, admixture, bottlenecks, gene flow, genetic drift, inbreeding, and selection (Slatkin, 2008). As a consequence, LD across the genome can vary within and between populations.

Thus, another approach to detect genomic selection signatures is based on statistical comparisons of atypical LD patterns at specific haplotypes of certain genomic regions that are inconsistent with the neutral evolution model (Mueller, 2004). This approach has been used in numerous studies to detect selection signatures in humans and in domesticated species (Sabeti et al., 2002; Voight et al., 2006; Hayes et al., 2008). These studies are based on the concept that in a large population, a neutral variant, which by definition is not under selection, will take many generations to become fixed or lost. Recombination and the passing of generations act with stronger intensity, and therefore, LD around these neutral alleles erodes quickly, leaving a smaller surrounding haplotype (Kimura, 1983; Nielsen et al., 2005b; Sabeti et al., 2006).

Conversely, alleles under positive or balanced selection carry other linked alleles with them, generating increased LD in the genomic region, as described for the hitchhiking effect (Smith and Haigh, 1974). LD between these alleles is slowly eroded, such that the adjacent haplotype is longer than expected by chance (Sabeti et al., 2002). Thus, large haplotypes reflect positive selection. This forms the basis of the EHH statistic (“extended haplotype homozygosity”) suggested by Sabeti et al. (2002), which is defined as the probability that two randomly selected chromosomes carrying the core haplotype are identical by descent, and also measures the decay of haplotype homozygosity as a function of the distance. EHH allows for identification of regions with atypical frequencies of extended haplotypes and has been effectively used to detect signatures of recent positive selection within a population (Tang et al., 2004; Walsh et al., 2006).

Voight et al. (2006) developed the statistic |*iHS*| or “integrated Haplotype Score” which allows to compare the area under the curve of EHH distribution between ancestral and derived alleles. This approach is based on the fact that the EHH area of an allele under selection will be greater than that of a neutral allele; therefore, the integral of EHH captures this effect. *iHS* corresponds to a standardized ratio between the areas under the curve of ancestral and derived alleles, which is equal to 0 when the EHH decay is similar for both types of alleles. A negative *iHS* value near -1 indicates extended haplotype around a derived allele, whereas positive values near one indicate extended haplotype around an ancestral allele.

The *iHS* statistic is more sensitive for detecting rapid increases in frequencies of the derived allele produced by selection. However, it cannot detect selection signatures resulting from complete or nearly complete fixation of a beneficial allele in the population, and therefore cannot detect a significant fraction of variants under positive selection (Qanbari et al., 2011). For this reason, Tang et al. (2007) reported a new method involving comparison of *EHH* at the same site, but between populations, i.e., an approach based on the genetic diversity among divergent populations. These statistics are called site-specific *EHH* (*EHHS*); the area under the *EHHS* curve (*iES*); and the standardized ratio of *iES* between two populations (Hellmann et al., 2003), which reflect haplotype variation among populations. The search for selection signatures from EHH statistical derivatives has been performed in several species such as cattle (Qanbari et al., 2011), poultry (Li et al., 2012; Zhang et al., 2012), swine (Ai et al., 2013) and humans (Sabeti et al., 2007).

#### Index of population differentiation

The *F_ST_* (Wright, 1951) is a statistical measure of genetic variation due to differences in allele frequencies between and within populations (Holsinger and Weir, 2009). The *F_ST_* statistic has been one of the most widely used methods for detecting genomic regions that have been under selection (Gianola et al., 2010; Qanbari et al., 2011). The *F_ST_* for a locus that has been selected in one population but not another will be higher than in other loci not affected by selection, where genetic diversity is mainly caused by genetic drift (Holsinger and Weir, 2009). Genetic drift affects all loci in the genome similarly; however, loci under selection often behave differently and therefore may present atypical patterns of variation. These atypical patterns can be determined by genotyping, for example, a large number of single nucleotide polymorphisms (SNP) throughout the whole genome, where loci influenced by selection may be identified by deviations from the empirical distribution of *F_ST_* statistic (Cavalli-Sforza, 1966; Akey et al., 2002). That is, relative to a neutral model, outliers with value below a certain level suggest the effect of balanced selection, while outliers with values above a certain level are indicative of directional selection.

Various estimates of the *F_ST_* statistic have been developed and applied in a number of studies to search for selection signatures (Akey et al., 2002; Hayes et al., 2009; Amaral et al., 2011). However, although the outlier approach may be effective in identifying genes under selection, it poses several challenges, such as susceptibility to genotyping errors, population stratification, and false positives, as well as variations in mutation rate and low sensitivity (Narum and Hess, 2011). It is also well known that the outlier detection methods have limited power to detect disruptive selection (Beaumont and Balding, 2004) and weak forms of divergent selection (Wright and Gaut, 2005).

### TESTS BASED ON DIFFERENCES BETWEEN SPECIES

The statistical methodology to detect selection signatures by comparing information between species relies on the fact that genomic substitutions in coding regions are present in two forms: non-synonymous mutations (d_n_), which can lead to the replacement of amino acids in the resulting proteins, and synonymous mutations (d_s_), which do not cause amino acid substitution because of the redundancy of the genetic code (Nielsen, 2005; Biswas and Akey, 2006).

The d_n_/d_s_ ratio provides information about evolutionary forces acting upon a particular gene. For example, at loci under neutrality, the d_n_/d_s_ ratio will be equal to 1. Genes subject to functional limitations, such that a non-synonymous substitution is detrimental, will tend to be eliminated from the population by negative selection; therefore, d_n_/d_s_ <1. Conversely, an excess of non-synonymous mutations over synonymous mutations (d_n_/d_s_ >1) provides evidence for the action of positive selection in favor of non-synonymous substitution, which could provide a comparative advantage at the protein level (Nielsen et al., 2005a).

Based on these concepts, several studies have detected selection in many genes and organisms, such as genes related to immune response (Endo et al., 1996; Hughes, 1997; Sawyer et al., 2004), viral receptor genes (Fitch et al., 1997; Nielsen and Yang, 1998; Bush et al., 1999), genes associated with fertility (Swanson et al., 2001, 2003), and genes involved in sensory perception and smell in humans (Gilad et al., 2000).

### TESTS THAT USE INFORMATION WITHIN AND BETWEEN SPECIES

The neutral theory of molecular evolution indicates that genomic regions that evolve rapidly and, thus, have high divergence between species, will also show high levels of polymorphisms within species. The Hudson–Kreitman–Aguade (HKA) test compares the level of polymorphisms within each species and observed divergence between related species for two or more loci. The test can determine if it is likely that the observed difference is due to neutral or adaptive evolution (Hudson et al., 1987). The HKA test is the precursor to the McDonald–Kreitman test (Howe et al., 2013), which compares synonymous (P_S_) and non-synonymous (P_N_) mutations at a specific locus that are polymorphic within a species and synonymous (D_S_) and non-synonymous (D_N_) mutations that are fixed between species. Under neutrality, the ratios between P_N_/P_S_ and D_N_/D_S_ should be the same, while positive selection leads to increased divergence of synonymous substitutions (D_N_/D_S_ > P_N_/P_S_; McDonald and Kreitman, 1991).

## GENOMIC RESOURCES IN FISH

In recent decades, the development of DNA markers has greatly contributed to the study of animal genetics. DNA markers allow us to observe and exploit variation across the genome of an individual (Liu and Cordes, 2004; Tier, 2010).

In fish, a wide range of DNA markers have been used, including amplified fragment length polymorphisms (AFLP), random amplified polymorphic DNA (RAPD), sequence tagged sites (STS), variable number of tandem repeats (VNTR), microsatellites or simple sequence repeats (SSR), SNP, and expressed sequence tags (EST; Liu, 2007). Currently, with the development of high-throughput sequencing technologies many gigabases of nucleotide sequences can be generated in a short period of time, and many SNP and other polymorphisms can be detected using bioinformatics methods (Liu, 2011). These techniques provide an affordable and reliable scale of DNA sequencing in several organisms (Mardis, 2008). They are extensively used in *de novo* sequencing, quantification of gene expression by RNA-seq (“RNA sequencing”; Wang et al., 2009), massive identification of SNP markers using RAD-sequencing (“restriction site associated DNA sequencing”; Rowe et al., 2011), and population genomics studies (Hohenlohe et al., 2010; De Wit et al., 2012).

Although teleost fish are the largest group of vertebrates (about 27,000 species), they are underrepresented in genome sequencing projects (Spaink et al., 2013). **Table 1** shows some of the species that have undergone genome sequencing projects to date.

**Table 1:** Sequenced genomes available in fish as of 2013.

Order	Family	Species	Common name	Size estimation (Mb)	Chromosomes	Scaffolds	Status	Reference
Anguilliformes	Anguillidae	*Anguilla anguilla*	European eel	1,000	**–**	–	Scaffolds or contigs	Henkel et al. (2012a)
		*Anguilla japonica*	Japanese eel	1,150	**–**	323,776	Scaffolds or contigs	Henkel et al. (2012b)
Beloniformes	Adrianichthyidae	*Oryzias latipes*	Japanese rice fish	585.33	24	82,496	Chromosomes	Kasahara et al. (2007)
Characiformes	Characidae	*Asty*an*ax mexicanus*	Mexican Tetra	964.31	–	10,735	Scaffolds or contigs	Hinaux et al. (2013)
Coelacanthiformes	Latimeriidae	*Latimeria chalumnae*	African coelacanth	2,183.72	–	22,818	Scaffolds or contigs	Amemiya et al. (2013)
Cypriniformes	Cyprinidae	*Danio rerio*	Zebra fish	1,412.47	25	4,560	Chromosomes	Hinaux et al. (2013), Howe et al. (2013)
		*Cyprinus carpio*	Common carp	875	25	–	Chromosomes	Xu et al. (2014)
Cyprinodontiformes	Nothobranchiidae	*Nothobranchius furzeri*	Turquoise killifish	1,500	–	5,299	Scaffolds or contigs	Reichwald et al. (2009)
		*Nothobra*nc*hius kuhntae*	Beira killifish	–	–	5,934	Scaffolds or contigs	
	Poeciliidae	*Xiphophorus maculatus*	Southern platyfish	652.84		20,640	Scaffolds or contigs	Schartl et al. (2013)
Gadiformes	Gadidae	*Gadus morhua*	Atlantic cod	608.29	–	427,427	Scaffolds or contigs	Star et al. (2011)
Gasterosteiformes	Gasterosteidae	*Gasterosteus aculeatus*	Three-spined stickleback	446.62	–	–	Scaffolds or contigs	
Lepisosteiformes	Lepisosteidae	*Lepisosteus oculatus*	Spotted gar	945.86	29	2,105	Chromosomes	
Perciformes	Cichlidae	*Oreochromis niloticus*	Nile tilapia	816.12	–	5,901	Scaffolds or contigs	
		*Pundamilia nyererei*	Python island	698.8	–	7,236	Scaffolds or contigs	
		*Rhamphochromis esox*	–	1,100	–	55,751	Scaffolds or contigs	Loh et al. (2008)
		*Mchenga conophoros*	–	1,100	–	61,923	Scaffolds or contigs	Loh et al. (2008)
		*Melanochromis auratus*	Golden mbuna	1,100	–	63,297	Scaffolds or contigs	Loh et al. (2008)
		*Neolamprologus brichardi*	Princess Burundi	685.96	–	9,098	Scaffolds or contigs	
		*Haplochromis burtoni*	–	698.98	–	8,001	Scaffolds or contigs	
		*Labeotropheus fuelleborni*	Blue mbuna	1,100	–	58,245	Scaffolds or contigs	Loh et al. (2008)
		*Maylandia zebra*	Zebra mbuna	1,100	–	65,094	Scaffolds or contigs	Loh et al. (2008)
Perciformes	Moronidae	*Dicentr*ar*chus labrax*	European sea bass	98.25	–	–	Scaffolds or contigs	
Pleuronectiformes	Cynoglossidae	*Cynoglossus semilaevis*	Flatfish	477	21	80,677	Chromosomes	Chen et al. (2014)
Salmoniformes	Salmonidae	*Oncorhynchus mykiss*	Rainbow trout	1,877.5	29	79,941	Chromosomes	Berthelot et al. (2014)
		*Salmo salar*	Atlantic salmon	2,435.31	29	843,055	Chromosomes	Davidson et al. (2010)
Tetraodontiformes	Tetraodontidae	*Takifugu flavidus*	Sansaifugu	314.95	–	34,332	Scaffolds or contigs	
		*Takifugu rubripes*	Pufferfish	281.57	22	7091	Chromosomes	Aparicio et al. (2002)
		*Tetraodon nigroviridis*	Green spotted puffer	308.45	–	–	Scaffolds or contigs

Extracted and modified from Spaink et al. (2013). The terms scaffolds or contigs indicate that the genome of the species has been partially sequenced, and the term chromosome indicates that sequencing has been anchored to the existing physical map of the species.

## SELECTION SIGNATURES IN FISH

In fish, studies aimed at detecting selection signatures are performed mainly in the context of molecular ecology disciplines. Most of them have been limited to a low level of resolution and restricted to specific genomic regions.

### MODEL FISH SPECIES

Using SNP markers from ESTs, loci with outlier *F_ST_* values were identified in wild populations of zebrafish (*Danio rerio*), suggesting directional selection in genes associated with energy metabolism, homeostasis regulation, and signal transduction, which could be associated with local adaptation among different populations. Further, evidence was found to suggest balanced selection of the gene encoding the receptor for the NS1A influenza virus (Whiteley et al., 2011). In the same study, outlier *F_ST_* values were found for loci in laboratory strains related to oxidoreductase activity, chromatin condensation, immune response, and induction of apoptosis, among other processes, which could be associated with the domestication process of cultured strains (Whiteley et al., 2011).

### CICHLIDS

Keller et al. (2013) detected outlier SNP patterns between five cichlid species from the Lake Victoria area in East Africa, identifying signatures of divergent selection between the two genera that include these species. These selection signals were associated with male color, depth distribution, feeding patterns, and morphological traits that distinguish the genera. Moreover, evidence has been found to suggest selection in the homeobox genes (*dlx*) involved in the development of the nervous system, the craniofacial skeleton, and the formation of connective tissue and appendages (Diepeveen et al., 2013).

### SALMONIDS

In lake whitefish (*Coregonus clupeaformis*), a fish of the salmon family distributed along northern Alaska and all of Canada, 24 loci were identified that revealed selection signatures associated with QTL of certain adaptive traits such as natatorium behavior, growth rate, morphology, and reproduction characters (Rogers and Bernatchez, 2007).

In Atlantic salmon, Vasemägi et al. (2012) used microsatellite markers and SNP to locate 10 genomic regions showing signatures of directional selection related to characteristics such as growth rate and morphology. Martinez et al. (2013) found strong evidence of selection in a microsatellite marker on chromosome 3, which harbored QTL for body weight. Furthermore, there is evidence that genes associated with immune response have been subject to greater selection pressure compared with other regions of the genome (Tonteri et al., 2010; Portnoy et al., 2014). Other studies in genera *Oncorhynchus*, *Salmo*, and *Salvelinus* have revealed signatures of balanced selection for genes of the major histocompatibility complex IIB (Aguilar and Garza, 2007; Limborg et al., 2012). In brown trout, analysis with markers linked to genes related to the immune response showed evidence of having been subjected to selection (Jensen et al., 2008). Finally, evidence was found in both brown trout and sockeye salmon of disruptive selection at two loci within the major histocompatibility complex IIB (Hansen et al., 2010; Gomez-Uchida et al., 2011; Meier et al., 2011).

### OTHER FAMILES

In guppies (*Poecilia reticulata*), outlier *F_ST_* values suggest that between 3.5 and 6.5% of SNP markers are under directional selection. Some of these loci are near QTL associated with ornamental traits, and they are also in EST (Willing et al., 2010).

In Atlantic cod in 1960, Sick detected evidence of selection in the locus encoding Hemoglobin (*Hb I*) and the *Pan I* locus, which encodes a protein related to the neuroendocrine system and has recently been associated with vesicle transport in adipocytes (Pogson, 2001). In the same species, Moen et al. (2008) identified 29 SNP with outlier *F_ST_*, suggesting that these loci are or have been under selection. These loci were found in genes involved in muscle contraction, immune response, and production of ribosomal proteins. Moreover, Nielsen (Nielsen et al., 2009) found evidence of directional selection for local adaptation to various environmental conditions, such as loci with outlier *F_ST_* values associated with genes involved in the production of proteins for thermal shock (*Hsp90*), determination of sexual behavior (*Aromatasa*), and formation of photoreceptor cells for perception of light (*rhodopsin*).

In stickleback (*Gasterosteus aculeatus*), a fish of the *Gasterosteidae* family, studies using microsatellite markers to assess genetic diversity among marine and freshwater populations revealed evidence of directional selection that might be associated with adaptation of certain populations to freshwater environments (Mäkinen et al., 2008).

## FUTURE DIRECTIONS

The search for and detection of genomic signatures produced by selection has provided valuable information that contributes to the understanding of evolutionary forces affecting the genome and gene functions that control phenotypes of biological and economic interest (Nielsen, 2005; Nielsen et al., 2007).

Some fish species provide the great advantage of simultaneous availability as both a wild and a cultivated population. Additionally, these species have unique characteristics in terms of population structure and intra-specific adaptive divergence, mainly due to the diversity of environmental conditions that fish populations inhabit, resulting in populations that exhibit characteristics of strong local adaptation. Comparative studies among these populations would provide benefits in terms of elucidating the effects of selective processes and recent domestication events, which could improve the understanding regarding the impact of the interaction between domesticated and wild populations, the identification of genetic factors involved in economically important traits for aquaculture and unravelling the actual phenotypic variation within and between fish populations.

In domesticated species, the main motivation behind the search for selection signatures lies in the possibility of finding genes or genomic regions associated with traits of economic interest. The development of next-generation sequencing technologies and high-throughput genotyping has made it possible to investigate the effect of selective pressures on genome variation in several domesticated species. In cattle and sheep, researchers have detected selection signatures associated with carcass yield traits, tail fat deposition, dairy traits (Moradi et al., 2012; Rothammer et al., 2013), reproductive traits (Gautier and Naves, 2011; Qanbari et al., 2011), immune response (Gautier and Naves, 2011), coat color, and horn development (Druet et al., 2013), among other characters of interest. Also, in swine, selection signatures have been identified in genomic regions associated with traits such as coat color, ear morphology, reproductive characteristics, and fat deposition (Wilkinson et al., 2013). In chickens, researchers have identified selection signatures associated with eggshell hardness and immune system characteristics (Qanbari et al., 2012), to mention just a few examples. These studies may provide a basis for conducting similar research that allows for investigation of the genomic regions affected by the processes of domestication and natural or artificial selection in fish populations, allowing for discovery of new genes that underlie phenotypic traits of interest and understanding processes relevant for conservation purposes.

There is currently little genomic information for fish species as compared to humans or domesticated animals. This is one of the reasons why selection signatures studies have been conducted in only a few species and generally limited to a low level of genomic coverage (Vasemägi et al., 2005, 2012). However, recent advances in genomic technologies, including high quality reference genome sequences, construction of genetic maps, and development of high-density SNP arrays are paving the way for systematic study of genetic variation in these species.

The development and application of next-generation sequencing approaches will represent a powerful strategy to improve the resolution and accuracy when detecting regions under selection in several species. This may lead to determination of the causative genetic factors involved in several biological aspects of aquaculture species. However, the application of these results in the aquaculture development requires further studies aiming at determining effective and practical applications of this technology. Candidate disciplines to be benefited from the discovery of selected regions using next-generation sequencing are, for example, genetic improvement, vaccine and pharmaceutical development and fish nutrition.

## CONCLUSION

The development of genomic methodologies has contributed greatly to the study of genetic variation between and within species. High-resolution studies at the level of the whole genome can identify selection signatures explaining phenotypic variation between and within populations, and therefore potentially identify genetic variants underlying characteristics of biological and economic interest.

Although the application and utility of these techniques in aquaculture species has been limited by a lack of genomic information, there is a great potential for conducting such studies, especially in species for which there are genome sequencing projects and high-density molecular markers platforms availability.

## Conflict of Interest Statement

The authors declare that the research was conducted in the absence of any commercial or financial relationships that could be construed as a potential conflict of interest.
